# *RACK1 *genes regulate plant development with unequal genetic redundancy in *Arabidopsis*

**DOI:** 10.1186/1471-2229-8-108

**Published:** 2008-10-23

**Authors:** Jianjun Guo, Jin-Gui Chen

**Affiliations:** 1 Department of Botany, University of British Columbia, Vancouver, BC V6T 1Z4, Canada

## Abstract

**Background:**

RACK1 is a versatile scaffold protein in mammals, regulating diverse developmental processes. Unlike in non-plant organisms where RACK1 is encoded by a single gene, Arabidopsis genome contains three *RACK1 *homologous genes, designated as *RACK1A, RACK1B *and *RACK1C*, respectively. Previous studies indicated that the loss-of-function alleles of *RACK1A *displayed multiple defects in plant development. However, the functions of *RACK1B *and *RACK1C *remain elusive. Further, the relationships between three *RACK1 *homologous genes are unknown.

**Results:**

We isolated mutant alleles with loss-of-function mutations in *RACK1B *and *RACK1C*, and examined the impact of these mutations on plant development. We found that unlike in *RACK1A*, loss-of-function mutations in *RACK1B *or *RACK1C *do not confer apparent defects in plant development, including rosette leaf production and root development. Analyses of *rack1a*, *rack1b *and *rack1c *double and triple mutants, however, revealed that *rack1b *and *rack1c *can enhance the rack1a mutant's developmental defects, and an extreme developmental defect and lethality were observed in rack1a rack1b rack1c triple mutant. Complementation studies indicated that RACK1B and RACK1C are in principle functionally equivalent to *RACK1A*. Gene expression studies indicated that three *RACK1 *genes display similar expression patterns but are expressed at different levels. Further, *RACK1 *genes positively regulate each other's expression.

**Conclusion:**

These results suggested that *RACK1 *genes are critical regulators of plant development and that *RACK1 *genes function in an unequally redundant manner. Both the difference in *RACK1 *gene expression level and the cross-regulation are likely the molecular determinants of their unequal genetic redundancy.

## Background

Receptor for activated C kinase 1 (RACK1) is a seven tryptophan-aspartic acid-domain (WD40) repeat-containing protein, and was originally identified as an anchoring protein for protein kinase C (PKC) in mammals, shuttling the activated enzyme to different subcellular sites [1,2]. Structurally, RACK1 is similar to the heterotrimeric G-protein β subunit (Gβ) which has a seven-bladed propeller structure with one WD40 unit constituting each blade (reviewed in [3,4]). Increasing evidence suggests that in addition to binding the activated PKC, mammalian RACK1 functions as a scaffold protein by physically interacting with many other proteins and facilitating their interactions. It has been shown that RACK1 plays regulatory roles in diverse developmental and physiological responses, including cell cycle control, cell movement and growth, immune response, and neural responses in mammals (reviewed in [3,4]). Therefore, RACK1 is now viewed as a versatile scaffold protein, serving as a nexus for multiple signal transduction pathways.

Although not recognized as such, the first plant *RACK1 *gene was cloned from tobacco BY-2 cells as an auxin (2,4-dichlorophenoxyacetic acid, 2,4-D) inducible gene, *arcA *[5]. Subsequently, the amino acid sequence homologues of RACK1 were found in all plant species examined (reviewed in [6]). Earlier studies based on gene expression and induction analysis implied that plant RACK1 may have a role in hormone-mediated cell division [5,7], UV and salicylic acid responses [8]. In rice, RACK1, named RWD [9], was found to be one of the seven proteins whose expressions were down-regulated in *d1 *mutant, a loss-of-function allele of rice heterotrimeric G-protein α subunit [10]. Further, rice RACK1 protein was induced by abscisic acid (ABA) in imbibed wild-type seeds, but not in *d1 *mutant seeds. It was proposed that RACK1 may play a role in rice embryogenesis and germination [10]. Furthermore, recently, it has been demonstrated that RACK1 proteins are key regulators of innate immunity by interacting with multiple proteins in the Rac1 immune complex in rice [11]. In Arabidopsis, RACK1 proteins have been found to be associated with the subunits of ribosomes [12,13], but no signaling proteins have been identified to interact with Arabidopsis RACK1 proteins.

**Figure 1:**
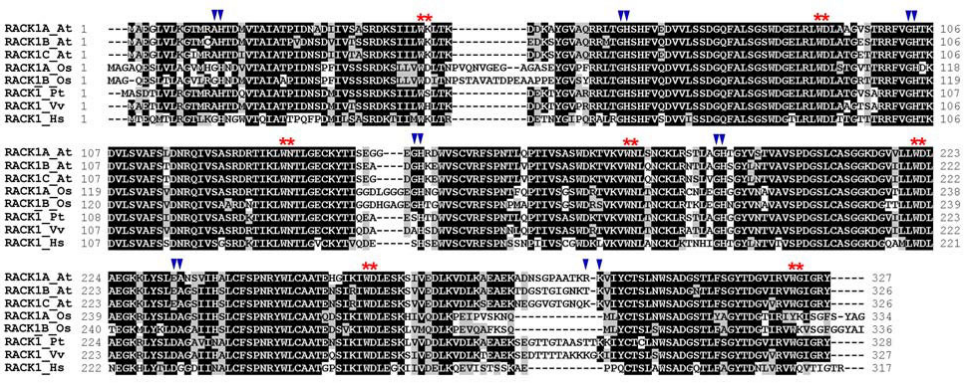
**Multiple amino acid sequence alignment of RACK1 in plants and in humans**. The amino acid sequences were aligned by CLUSTALW multiple alignment of BioEdit Sequence Alignment Editor . Amino acids that are identical or similar are shaded with black or gray, respectively. Gaps are shown as dashed lines. The proteins aligned are (name of species and accession number in parentheses): RACK1A_At (*Arabidopsis thaliana*, NP_173248), RACK1B_At (*Arabidopsis thaliana*, NP_175296), RACK1C_At (*Arabidopsis thaliana*, NP_188441), RACK1A_Os (*Oryza sativa*, NP_001043910), RACK1B_Os (*Oryza sativa*, NP_001056254), RACK1_Pt (*Populus trichocarpa*, ABK92879), RACK1 _Vv (*Vitis vinifera*, CAN61810), and RACK1_Hs (*Homo sapiens*, NP_006089). The positions of GH and WD dipeptides in each WD40 repeat are indicated by triangles and asterisks, respectively, on the top of residues. The positions for WD repeat domains were obtained from the SMART database .

Structurally, RACK1 proteins in plants are similar to those in mammals, containing a seven-bladed β-propeller [14]. However, analysis of RACK1 proteins in plants and in non-plant organisms revealed an important feature of plant RACK1 proteins: some plants have more than one *RACK1 *genes, in contrast to the single copy of *RACK1 *gene in non-plant organisms. For example, the sequenced genomes of rice (*Oryza sativa*) and Arabidopsis (*Arabidopsis thaliana*) contain two and three *RACK1 *homologous genes, respectively (Figure 1). The three RACK1 proteins encoded by the Arabidopsis genome were designated as RACK1A, RACK1B and RACK1C, respectively [15]. Previously, we provided evidence that *RACK1A *mediates multiple hormone responses and developmental processes [15]. However, the functions of the other two Arabidopsis *RACK1 *genes, *RACK1B *and *RACK1C*, and the relationship between Arabidopsis *RACK1 *genes remain unknown. Here we demonstrate that although *RACK1B *and *RACK1C *genes are likely dispensable, they still contribute significantly to the *RACK1A*-regulated developmental processes in Arabidopsis. We provide evidence that the difference in the gene expression level and the cross-regulation are likely the molecular determinants of unequal genetic redundancy of *RACK1 *genes in regulating plant development.

## Results

### T-DNA insertional mutants of *RACK1B *and *RACK1C*

**Figure 2:**
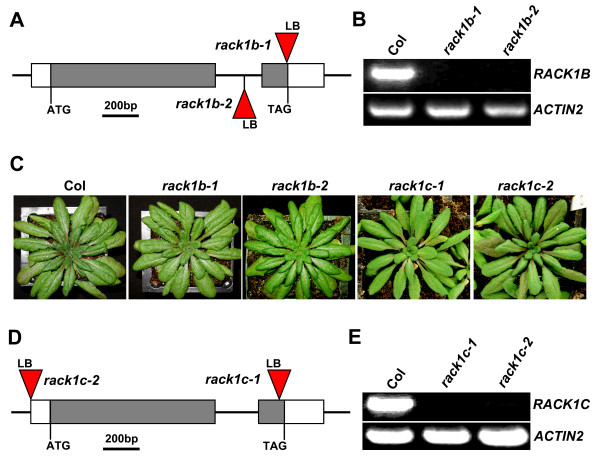
**T-DNA insertional mutants of *RACK1B *and *RACK1C***. (**A**) A diagram to illustrate the T-DNA insertion sites in *rack1b-1 *and *rack1b-2 *mutants. (**B**) RT-PCR analysis of *RACK1B *transcript in *rack1b *mutants. *RACK1B*-specific primers that amplify the full-length transcript of *RACK1B *in wild-type (Col) were used. (**C**) The rosette morphology of *rack1b *and *rack1c *mutants. Shown are plants grown 48 days under 10/14 h photoperiod. (**D**) A diagram to illustrate the T-DNA insertion sites in *rack1c-1 *and *rack1c-2 *mutants. (**E**) RT-PCR analysis of *RACK1C *transcript in *rack1c *mutants.*RACK1C*-specific primers that amplify the full-length transcript of *RACK1C *in Col were used. Gray boxes in (A) and (D) represent coding regions and white boxes represent 5'-UTR and 3'-UTR regions. The T-DNA inserts are not drawn to scale. LB, T-DNA left border. Total RNA isolated from 10 d-old, light-grown seedlings was used for RT-PCR analysis in (B) and (E). RT-PCR was performed with 30 cycles. The expression of *ACTIN2 *was used as a control.

Arabidopsis genome contains three *RACK1 *homologous genes, designated as *RACK1A*, *RACK1B *and *RACK1C*, respectively [15]. Within the *RACK1 *gene family, mutant alleles for only *RACK1A *have been reported previously [15]. We report here the isolation and characterization of *rack1b *and *rack1c *mutant alleles. By searching the Salk Institute sequence-indexed insertion mutant collection , we obtained two independent T-DNA insertional alleles for each *RACK1 *gene. All alleles are in the Columbia (Col-0) ecotypic background. We designated the two mutant alleles for *RACK1B *as *rack1b-1 *and *rack1b-2*, respectively. In *rack1b-1 *allele, the T-DNA was inserted in the second exon of *RACK1B *gene, and in the *rack1b-2 *allele, the T-DNA was inserted in the first intron (Figure 2A). RT-PCR analysis indicated that the full-length transcript of *RACK1B *was absent in both alleles (Figure 2B), implying that they are likely loss-of-function alleles. Unlike *rack1a *mutants, *rack1b *mutants do not display any apparent developmental defects (Figure 2C). We designated the two mutant alleles for *RACK1C *as *rack1c-1 *and *rack1c-2*, respectively (Figure 2D). In *rack1c-1 *allele, the T-DNA was inserted in the second exon of *RACK1C *gene, and in the *rack1c-2 *allele, the T-DNA was inserted in the 5'-UTR region. RT-PCR analysis indicated that the full-length transcript of *RACK1C *was absent in both alleles (Figure 2E), implying that they are likely loss-of-function alleles. Similar to *rack1b *mutants but unlike *rack1a *mutants, *rack1c *mutants do not display any apparent defects in plant development (Figure 2C).

### Loss-of-function mutations in *RACK1B *and *RACK1C *enhance the developmental defects in rosette leaf production of rack1a mutant

**Figure 3:**
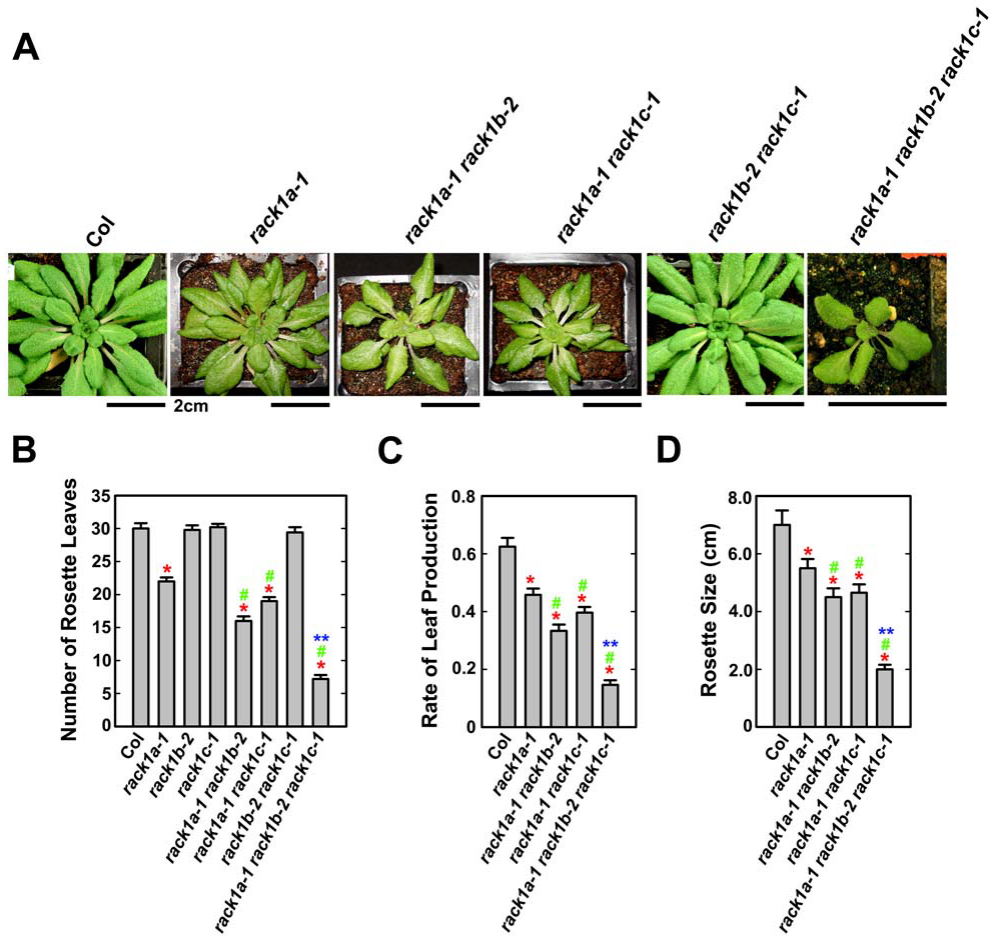
***rack1b-2 *and *rack1c-1 *mutations enhance the rosette leaf phenotype of *rack1a *mutants**. (**A**) The phenotype of *rack1 *mutants. Shown are plants grown for 48 days under 10/14 h photoperiod. Scale bars, 2 cm. (**B**) The number of rosette leaves of *rack1 *mutants. (**C**) The rate of rosette leaf production of *rack1 *mutants. The rate of rosette leaf production is expressed as the number of rosette leaves divided by the age of plants. (**D**) The size of rosette of *rack1 *mutants. The number of rosette leaves, the rate of rosette leaf production and the size of rosette were measured from plants grown for 48 d under 10/14 h photoperiod. Shown in (B) to (D) are the averages of at least four plants ± S.E. The same experiment was repeated twice with similar trends and the data from one experiment were presented. *, significant difference from Col, P < 0.05. #, significant difference from *rack1a *single mutant, P < 0,05. **, significant difference from *rack1a-1 rack1b-2 *double mutant, P < 0.05.

Previously, we showed that loss-of-function mutations in one member of Arabidopsis *RACK1 *gene family, *RACK1A*, resulted in multiple defects in plant development [15]. Because loss-of-function alleles of *RACK1B *and *RACK1C *did not display apparent defects in plant development, we wanted to test if mutations in *RACK1B *or *RACK1C *can enhance the developmental defects of *rack1a *mutants. Therefore, we generated *rack1a-1 rack1b-2 *and *rack1a-1 rack1c-1 *double mutants. One of the most dramatic phenotypes observed in *rack1a *single mutants was the reduced number of rosette leaves [15]. Therefore, we grew single and double mutants together with wild-type (Col) under identical, short-day conditions with 10/14 h photoperiod, counted the number of rosette leaves in double mutants, and compared it with Col and *rack1a-1 *single mutant. We found that while *rack1b-2 *and *rack1c-1 *single mutants produced wild-type number of rosette leaves, both *rack1b-2 *and *rack1c-1 *significantly enhanced the phenotype of reduced number of rosette leaves of *rack1a-1 *single mutants (Figure 3A, B). When plants were grown under 10/14 h photoperiod for 48 days, wild-type produced approximately 30 rosette leaves, whereas *rack1a-1 *single mutant produced 22 rosette leaves. Under these conditions, *rack1a-1 rack1b-2 *and *rack1a-1 rack1c-1 *double mutants only produced about 16 and 19 rosette leaves, respectively (Figure 3B). The rate of rosette leaf production was reduced approximately 27% and 14%, respectively, in *rack1a-1 rack1b-2 *and *rack1a-1 rack1c-1 *double mutants, compared with *rack1a-1 *single mutant (Figure 3C). We also examined the rosette size by measuring the diameter of rosette of each genotype. Similar to the situation of number of rosette leaves, the diameter of rosette was significantly reduced in *rack1a-1 *single mutant, compared with wild-type plants, and such reduction was further enhanced in *rack1a-1 rack1b-2 *and *rack1a-1 rack1c-1 *double mutants (Figure 3D). Interestingly, no synergistic effect was observed between *rack1b-2 *and *rack1c-1 *mutations. Statistically, *rack1b-2 rack1c-1 *double mutants phenocopied parental single mutants and displayed wild-type traits of these phenotypes (Figure 3A, B).

Subsequently, we generated *rack1a-1 rack1b-2 rack1c-1 *triple mutant. Very few triple mutants could survive in soil. For those survived, they were extremely slow in growth and development, and produced fewest rosette leaves and smallest rosette size among all genotypes examined (Figure 3A–D). Not surprisingly, the rate of rosette leaf production in the triple mutant was the slowest among all genotypes examined (Figure 3C). Because *rack1a-1 rack1b-2 rack1c-1 *triple mutants could not survive to maturity to produce seeds, these triple mutants were maintained in plants homozygous for the *rack1b-2 *and *rack1c-1 *loci and heterozygous for the *rack1a-1 *locus. Because *rack1b-2 rack1c-1 *double mutants had wild-type morphology whereas *rack1a-1 rack1b-2 rack1c-1 *had extreme pleiotropic phenotype, *rack1a-1 rack1b-2 rack1c-1 *triple mutants can be readily picked up from the segregating progeny of plants homozygous for the *rack1b-2 *and *rack1c-1 *loci and heterozygous for the *rack1a-1 *locus.

### Loss-of-function mutations in *RACK1B *and *RACK1C *enhance the defects in root development of rack1a mutant

**Figure 4:**
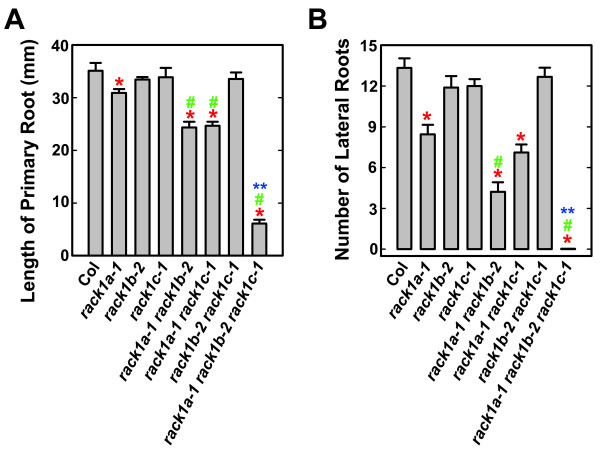
***rack1b-2 *and *rack1c-1 *mutations enhance the root phenotype of *rack1a *mutants**. (**A**) The length of primary root of *rack1 *mutants. (**B**) The number of lateral roots of *rack1 *mutants. The length of primary root and the number of lateral roots were measured from 10 d-old, light-grown seedlings (under 14/10 h photoperiod). Shown are the averages of at least 15 seedlings ± S.E. *, significant difference from Col, P < 0.05. #, significant difference from *rack1a *single mutant, P < 0.05. **, significant difference from *rack1a-1 rack1b-2 *double mutant, P < 0.05.

Genetic analysis indicated that loss-of-function mutations in *RACK1A *affect the production of rosette leaves, and that the effect of *rack1a-1 *mutation can be enhanced by the *rack1b-2 *or *rack1c-1 *mutation or both (Figure 3). We wanted to extend our analysis to non-aerial organs by examining the impact of these mutations on root development. We measured the length of primary root and counted the number of lateral root and used them as parameters of root development and root architecture. We found that the length of primary root of *rack1a-1 *mutant was slightly shorter than that of wild-type whereas *rack1b-2 *and *rack1c-1 *mutants had wild-type length of primary root (Figure 4A). The length of primary root was further shortened in *rack1a-1 rack1b-2 *and *rack1a-1 rack1c-1 *double mutants, compared with that in *rack1a-1 *single mutant (Figure 4A), indicating that *rack1b-2 *and *rack1c-1 *mutations can also enhance the effect of *rack1a-1 *mutation on primary root growth. Similar to the situation of primary root, *rack1a-1 *mutant produced fewer lateral roots than wild-type whereas *rack1b-2 *and *rack1c-1 *mutants had wild-type number of lateral roots (Figure 4B). As expected, *rack1b-2 *and *rack1c-1 *mutations enhanced the lateral root phenotype of *rack1a-1 *mutant (Figure 4B). Among all genotypes examined, the *rack1a-1 rack1b-2 rack1c-1 *triple mutant produced the shortest primary root and did not produce any lateral root under our assay conditions (Figure 4A, B).

### Genetic complementation of *rack1a *mutants by overexpressing *RACK1 *genes

**Figure 5:**
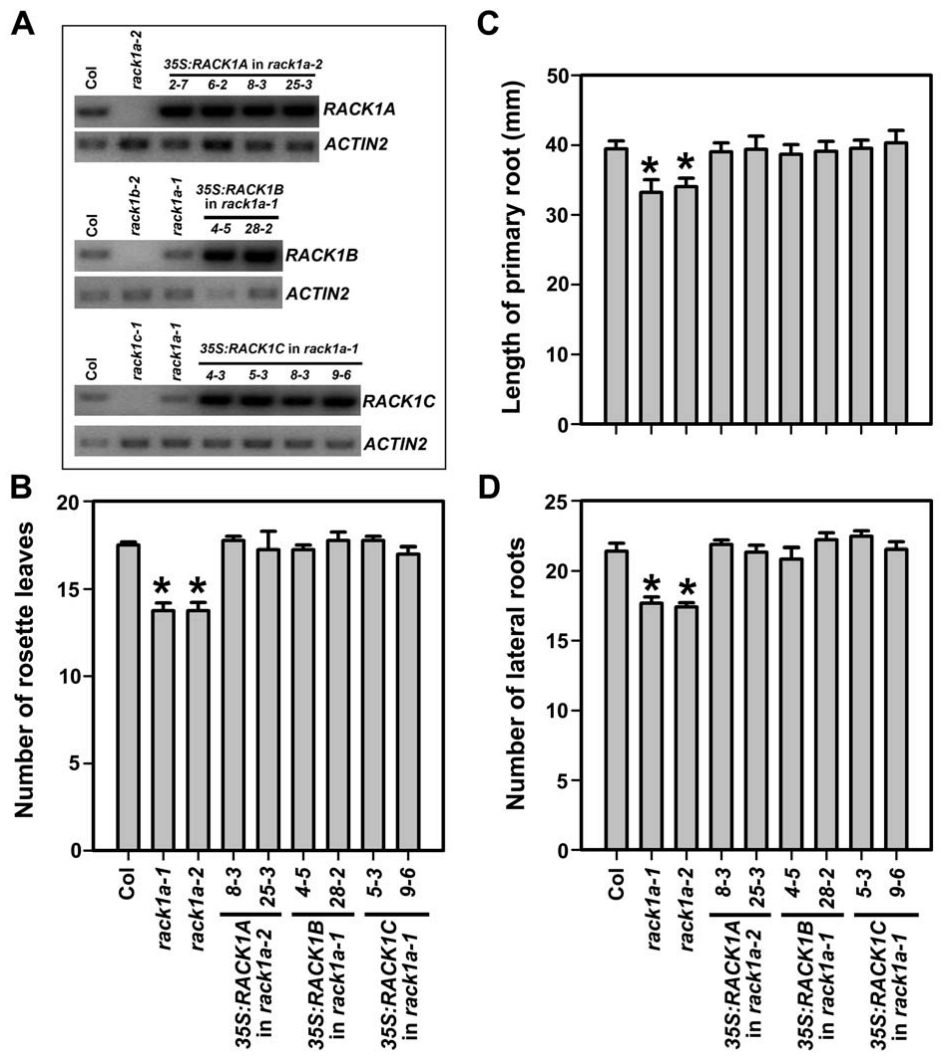
**The complementation of *rack1a *mutants by overexpression of *RACK1 *genes**. (**A**) RT-PCR analysis of the expression of *RACK1 *genes in transgenic lines. The transgenic lines *2-7*, *6-2*, *8-3 *and *25-3 *are *RACK1A *overexpressors in *rack1a-2 *mutants. The transgenic lines *4-5 *and *28-2 *are *RACK1B *overexpressors in *rack1a-1 *mutants. The transgenic lines *4-3*, *5-3*, *8-3 *and *9-6 *are *RACK1C *overexpressors in *rack1a-1 *mutants. RT-PCR was performed at 28 cycles. The expression of *ACTIN2 *was used as a control. (**B**) The number of rosette leaves in transgenic plants overexpressing individual *RACK1 *gene in *rack1a *mutant background. The number of rosette leaves was collected from plants grown for 37 d under 14/10 h photoperiod. Shown are the averages of number of rosette leaves from at least four plants ± S.E. (**C**) The length of primary root in transgenic plants overexpressing individual *RACK1 *gene in *rack1a *mutant background. The length of primary roots was measured from seedlings grown for 10 d under 14/10 h photoperiod. (**D**) The number of lateral roots in transgenic plants overexpressing individual *RACK1 *gene in *rack1a *mutant background. The number of lateral roots was counted from seedlings grown for 11 d under 14/10 h photoperiod. Shown in (C) and (D) are the averages of at least 20 seedlings ± S.E. *, significant difference from Col, P < 0.05.

Genetic analyses indicated that there is unequal genetic redundancy among three Arabidopsis *RACK1 *genes in regulating rosette leaf production and root development, and that *RACK1A *is likely a non-dispensable gene in this small gene family. Although *RACK1B *and *RACK1C *are likely dispensable, they still contribute significantly to the overall activity of *RACK1 *genes in regulating plant development, as revealed by the phenotypes of double and triple mutants. We wanted to further explore the mechanism of the unequal genetic redundancy of *RACK1 *genes. Firstly, because RACK1B and RACK1C are highly similar (about 90% identity) to RACK1A at the amino acid level (Figure 1), we wanted to test if RACK1B and RACK1C are in principle functionally equivalent to RACK1A. We reasoned that if RACK1B and RACK1C are indeed functionally equivalent to RACK1A, one would expect that overexpression of *RACK1B *or *RACK1C *complements the developmental defects of *rack1a *mutants. Therefore, we generated transgenic lines overexpressing *RACK1B *or *RACK1C *in the *rack1a *mutant background using the *CaMV 35S *promoter. As a control, we generated transgenic plants overexpressing *RACK1A *in *rack1a *mutant background. At least two independent transgenic lines were analyzed for each transformation. Overexpression of the transgene in these lines was confirmed by RT-PCR analysis (Figure 5A). We examined the same parameters described above, namely the number of rosette leaves, the length of primary root and the number of lateral roots in the transgenic lines overexpressing each *RACK1 *gene and compared them with those in Col and *rack1a *single mutants. As expected, overexpression of *RACK1A *fully complemented the mutant phenotype of *rack1a *mutant (Figure 5B–D). Similarly, we found that overexpression of *RACK1B *or *RACK1C *fully restored *rack1a *mutant to wild-type morphology, evident by the wild-type number of rosette leaves, wild-type length of primary root and wild-type number of lateral roots in transgenic lines (Figure 5B–D).

### Expression of Arabidopsis *RACK1 *genes

**Figure 6:**
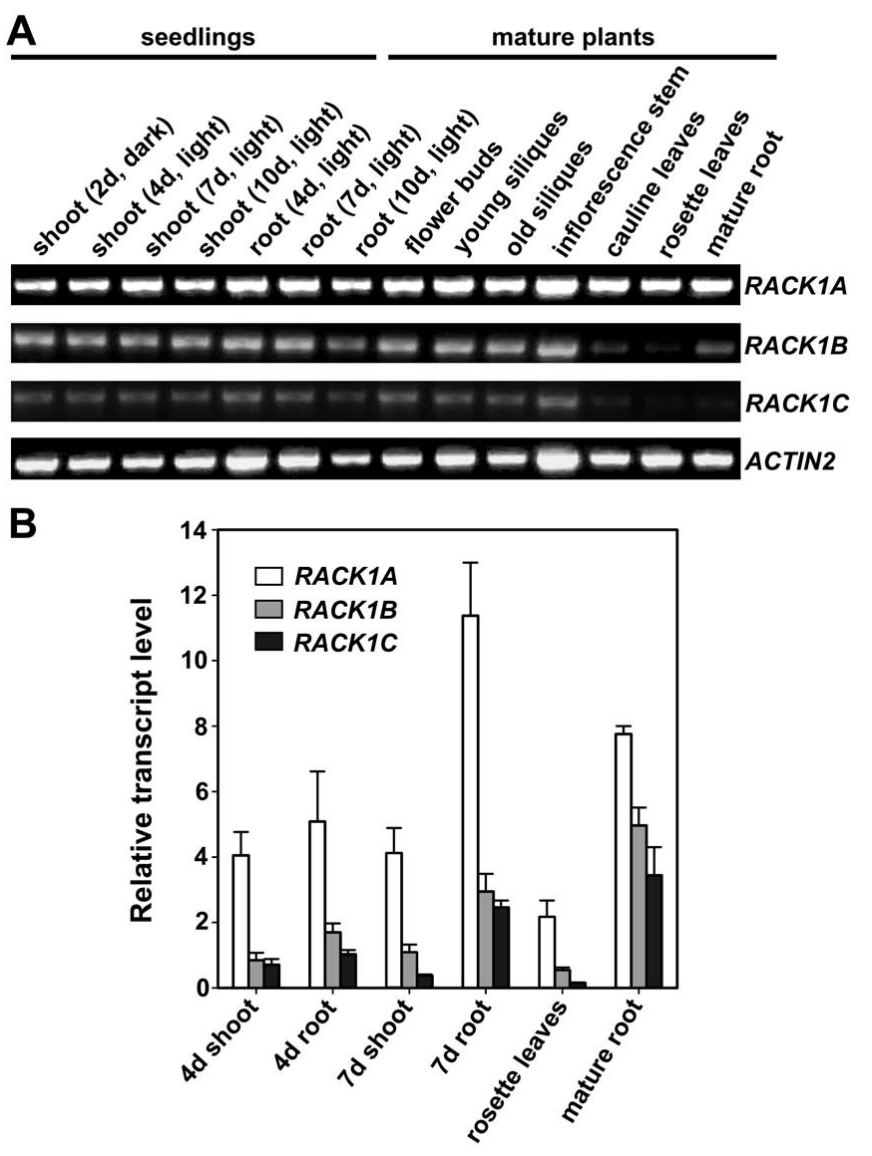
**The expression of *RACK1A*, *RACK1B *and *RACK1C *genes**. (**A**) RT-PCR analysis of the expression of *RACK1 *genes in various tissues and organs of young seedlings and mature plants. RT-PCR was performed at 30 cycles. The expression of *ACTIN2 *was used as a control. (**B**) Quantitative real-time PCR analysis of the transcript levels of *RACK1 *genes. The transcript level of each *RACK1 *gene was normalized against the transcript level of *ACTIN2 *in each sample. The relative transcript levels of *RACK1 *genes were compared to that of *RACK1C *in the roots of 4 d-old, light-grown seedlings (set as 1). Shown are the averages of three replicates ± S.D.

Because constitutive expression of *RACK1B *or *RACK1C *could efficiently complement *rack1a *mutant's developmental defects, these results implied that RACK1B and RACK1C are likely in principle functionally equivalent to RACK1A, and that the unequal genetic redundancy of *RACK1 *genes is likely due to the difference in their expression patterns or expression levels. Therefore, we sought additional evidence that would shed light on the relationship between *RACK1 *genes. We examined the expression patterns of *RACK1A*, *RACK1B *and *RACK1C *in various tissues and organs of young seedlings and mature plants by RT-PCR. We found that all three Arabidopsis *RACK1 *genes were expressed widely in all tissues examined (Figure 6A). These results are largely consistent with the results of analysis of *RACK1 *gene promoter:β-*glucuronidase *(*GUS*) transcriptional reporter lines [15]. By using RT-PCR, we noticed that in any given tissues or organs examined, the transcript level of three *RACK1 *genes were different, with a general trend of *RACK1A *> *RACK1B *> *RACK1C *(Figure 6A).

In order to quantify the difference in transcript level of *RACK1A*, *RACK1B *and *RACK1C *genes, we used quantitative real-time PCR to more accurately compare the transcript level of three *RACK1 *genes in different tissues and organs of wild-type Col plants. We selected the samples of shoots and roots of 4 d- and 7 d-old light-grown seedlings and rosette leaves and roots of mature plants for quantitative real-time PCR analysis. We found that consistent with the result of RT-PCR analysis, the transcript level of *RACK1C *was the lowest and that of *RACK1A *was the highest among three *RACK1 *genes, with a trend of *RACK1A *> *RACK1B *> *RACK1C *in all samples examined (Figure 6B). For example, the transcript level of *RACK1A *was about 5-fold higher than that of *RACK1C *in the roots of 4 d-old, light-grown seedlings (Figure 6B). In this sample, the transcript level of *RACK1B *was approximately 2-fold higher than that of *RACK1C*.

### Cross-regulation of *RACK1 *genes at the transcription level

**Figure 7:**
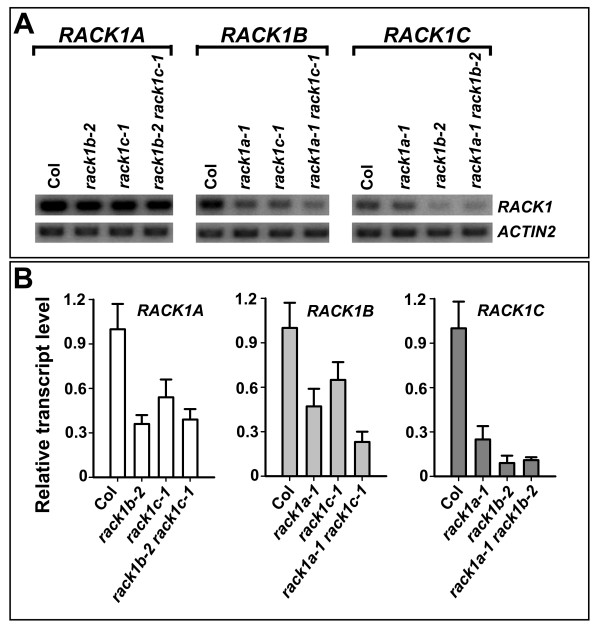
**The expression of *RACK1 *genes in *rack1a*, *rack1b *and *rack1c *single and double mutants**. (**A**) RT-PCR analysis of the expression of *RACK1 *genes in *rack1a*, *rack1b *and *rack1c *single and double mutants. RT-PCR was performed at 28 cycles. The expression of *ACTIN2 *was used as a control. (**B**) Quantitative real-time PCR analysis of the transcript level of *RACK1 *genes in *rack1a*, *rack1b *and *rack1c *single and double mutants. The transcript level of *RACK1 *genes was normalized against the transcript level of *ACTIN2 *in each sample. The relative transcript level of *RACK1 *genes in mutant backgrounds was compared with that in wild-type (Col) (set as 1). Shown are the averages of three replicates ± S.D.

The analysis of the expression patterns and transcript level of three *RACK1 *genes in various tissues and organs supported the view that the unequal genetic redundancy of *RACK1 *genes is likely due to the difference in the gene expression level. However, other possibilities may also exist. For example, as reviewed by Briggs et al. (2006), cross-regulation is another mechanism that attributes to the unequal genetic redundancy of some homologous genes [16]. Because RACK1A, RACK1B and RACK1C are approximately 90% identical to each other at the amino acid level, we were unable to obtain antibodies that can specifically recognize each RACK1 protein. Therefore, in this study, we examined the impact of loss-of-function mutations of each *RACK1 *gene on the transcription of the other two *RACK1 *genes. Further, we examined the impact of combination of loss-of-function mutations of two *RACK1 *genes on the transcription of the other *RACK1 *gene. Specifically, we examined the transcript level of *RACK1A *in *rack1b *and *rack1c *single and double mutants, the transcript level of *RACK1B *in *rack1a *and *rack1c *single and double mutants, and the transcript level of *RACK1C *in *rack1a *and *rack1b *single and double mutants, and compared with their transcript levels in wild-type. For this analysis, we used the 4.5 d-old, light-grown whole seedlings. By using RT-PCR, we noticed that the transcript level of *RACK1B *was reduced in *rack1a *and *rack1c *single and double mutants (Figure 7A). Similarly, the transcript level of *RACK1C *was reduced in *rack1a *and *rack1b *single and double mutants (Figure 7A). However, we did not observe a dramatic reduction of the transcript level of *RACK1A *in *rack1b *and *rack1c *single and double mutants, compared with that in wild-type (Figure 7A). Because the transcript level of *RACK1A *is the most abundant among three *RACK1 *homologous genes and the conditions used for RT-PCR (e.g. PCR at 28 cycles) may not allow us to visualize any differences in *RACK1A *transcript level among different samples, subsequently we used quantitative real-time PCR to more accurately compare the transcript level of three *RACK1 *genes in wild-type and mutants. We found that the transcript level of any given *RACK1 *gene was reduced in the loss-of-function alleles of each and both of the other two *RACK1 *genes (Figure 7B).

## Discussion

### Roles of *RACK1 *genes in plant development

*RACK1 *gene is evolutionarily conserved in diverse organisms. Although the research interest in RACK1 has grown exponentially since its discovery [1] and RACK1 is now viewed as a multi-functional, versatile scaffold protein in mammals and in yeasts (reviewed in [3,4]), the function of RACK1 in plants remains poorly understood. We are just starting to have some hints about its potential functions in plants. Preliminary analysis suggested RACK1 may mediate multiple hormone responses and developmental processes in Arabidopsis [15]. In this study, we focused on the two characteristic developmental defects of *rack1a *mutants, namely the reduction in rosette leaf production and the reduction in primary root growth and lateral root formation, to study the function of *RACK1B *and *RACK1C *and the genetic relationship between *RACK1 *homologous genes in plant development. We demonstrated that *RACK1 *genes are critical regulators of plant development and are essential for plant survival. Simultaneous disruption of the function of all three *RACK1 *genes results in lethality. Thanks to the unequal genetic redundancy of *RACK1 *genes, we are still able to study the role of *RACK1 *genes in plant development. The *rack1a *single mutants, *rack1a rack1b *and *rack1a rack1c *double mutants all display developmental defects and are viable. Therefore, these mutants can be treated as "weak alleles" of *rack1 *mutants. Now that we have identified *RACK1 *genes as critical regulators of plant development and all "weak alleles" of *rack1 *mutants are available, future studies should focus on the elucidation of the molecular mechanism by which *RACK1 *genes regulate plant development, including rosette leaf production, root growth and lateral root formation. Because *rack1a *mutants have also been shown to display altered responses to hormones [15], it remains unclear if the developmental defects observed in *rack1 *mutants are due to the altered responses to multiple hormones and if there is also unequal genetic redundancy of *RACK1 *genes in mediating hormone responses. This is a fertile area that is worth further investigation.

### Mechanism of unequal genetic redundancy of *RACK1 *genes

Genetic redundancy of homologous genes is thought to be due to gene duplication events during the evolution of the organism. Between homologous genes, genetic redundancy can be classified as full redundancy, partial redundancy, and unequal redundancy [16]. While full redundancy and partial redundancy have been documented in numerous cases, unequal genetic redundancy has just begun to be recognized as a common phenomenon of genetic relationship of homologous genes [16]. Unlike non-plant organisms whose genomes contain only a single *RACK1 *gene, some plant genomes contain more than one *RACK1 *genes (Figure 1). In particular, the Arabidopsis genome contains three *RACK1 *genes, which share the similar gene structure with two exons and one intron, and encode three highly similar proteins with approximately 90% identity at the amino acid level [15]. However, the relationship between three Arabidopsis *RACK1 *homologous genes has been unknown. Previously, we showed that loss-of-function mutation in one member of Arabidopsis *RACK1A *genes, *RACK1A*, conferred multiple defects in plant development [15]. Here we show that loss-of-function mutations in *RACK1B *or *RACK1C *do not confer apparent developmental defects (Figure 2). These results suggested that *RACK1B *and *RACK1C *are likely dispensable in plant development. However, we found that although *rack1b *and *rack1c *mutants displayed wild-type morphology, *rack1b *and *rack1c *can strongly enhance the developmental defects of *rack1a *mutants (Figure 3, Figure 4). These results suggested that *RACK1B *and *RACK1C *still contribute significantly to the overall activity of *RACK1 *genes. Because the significance of the *RACK1B *and *RACK1C *is determined via the mutants, not directly in the wild-type plants, it is also possible that in the wild-type plants, all the function of *RACK1 *genes is explicated by *RACK1A *with no contribution from *RACK1B *or *RACK1C *and the these latter can play a role only if RACK1A is not present (e.g. in the *rack1a *mutant). Nonetheless, the behaviors and relationship of *rack1 *mutants satisfy the key criteria for *RACK1 *genes being unequally redundant homologous genes [16].

The unequal genetic redundancy is caused by many factors. Among them, the difference in gene expression pattern, expression level and cross-regulation of homologous genes have been recognized as major determinants [16]. The unequal genetic redundancy of some homologous genes is mainly due to the difference in expression pattern and/or expression level. For example, *CAULIFLOWER *(*CAL*) is closely related in sequence to *APETALA1 *(*AP1*), but *AP1 *and *CAL *regulate the formation of floral meristem in an unequally redundant manner because *AP1 *is expressed at much higher level than *CAL *throughout sepal and petal development [17]. The unequal genetic redundancy of homologous genes can also be primarily due to the cross-regulation. For example,*LONG HYPOCOTYL 5 *(*HY5*) and its close homolog *HY5 HOMOLOG *(*HYH*), both of which are regulators of photomorphogenesis, are a pair of unequally redundant genes with similar expression patterns and levels [16,18], but a normal protein expression and activity of HYH was dependent on the presence of a functional HY5 [18].

In order to get insight into the mechanism of unequal genetic redundancy of three *RACK1 *genes, we examined each of these possibilities. Firstly, we showed that *RACK1B *and *RACK1C *are likely in principle functionally equivalent to *RACK1A*, because overexpression of either *RACK1B *or *RACK1C *under the constitutive *CaMV 35S *promoter fully complemented the developmental defects of *rack1a *mutants (Figure 5). Ideally, it would be advantageous to use the native *RACK1A *promoter to assess the extent of functional equivalency. Nonetheless, results from our complementation studies indicated that overexpression of *RACK1B *or *RACK1C *can restore *rack1a *mutant to wild-type equally well as overexpression of *RACK1A*, supporting the view that *RACK1B *and *RACK1C *likely function similarly as *RACK1A*. These results implied that the unequal genetic redundancy of *RACK1 *genes is likely due to the difference in gene expression pattern and/or expression level, rather than the difference in protein sequence or activity. To examine this possibility directly, we found that three *RACK1 *genes are widely expressed in various tissues and organs in young seedlings and in mature plants (Figure 6). However, *RACK1 *genes are expressed at different levels with a general trend of *RACK1A *> *RACK1B *> *RACK1C *in all tissues and organs examined (Figure 6). These results supported the view that the difference in gene expression level attributes to the unequal genetic redundancy of *RACK1 *genes in plant development. However, these results cannot rule out the possibility that the expression of each *RACK1 *gene may also be restricted to certain cell types. For example, BRL1 and BRL3 are homologous to BRI1, a receptor for brassinosteroid (BR), and function as BR receptors in vascular differentiation in Arabidopsis [19]. It was found that *BRI1 *is ubiquitously expressed in growing cells, but the expression of *BRL1 *and *BRL3 *is restricted to non-overlapping subsets of vascular cells. Future expression analysis at cell level (e.g. by in situ hybridization and reporter GFP analyses) may help address the possibility of cell type-specific expression of *RACK1 *genes.

We also explored the possibility of cross-regulation by examining the transcript level of each *RACK1 *gene in the loss-of-function alleles of each or both of the other two *RACK1 *genes. We found that the transcript level of any given *RACK1 *gene was reduced in the single or double mutants for the other two *RACK1 *genes (Figure 7). Therefore, both the difference in gene expression level and the cross-regulation contribute to the unequal genetic redundancy of *RACK1 *genes. Unlike *HY5 *and *HYH*, for which the expression of the duplicate gene (*HYH*) depends on the presence of the ancestral gene (*HY5*) [18], *RACK1 *homologous genes mutually depend on each other for reaching full expression, adding another level of complexity for the unequal genetic redundancy. The molecular basis of such mutual cross-regulation of *RACK1 *genes is presently unknown. It would be interesting to test if RACK1 proteins can work together in a complex, for instance, through homo- and hetero-dimerization.

## Conclusion

**Figure 8:**
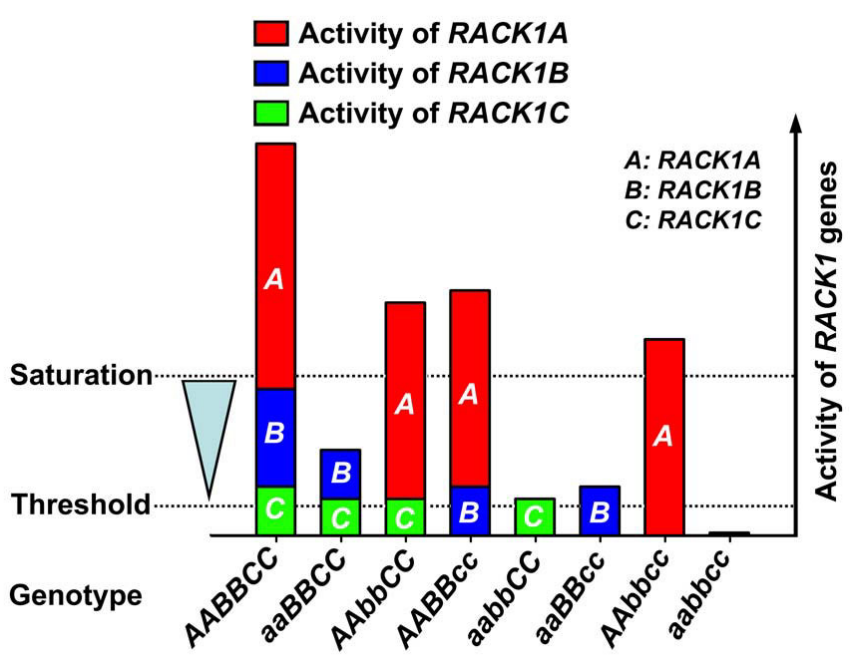
**The model of unequal genetic redundancy of *RACK1 *genes in regulating plant development**. Arabidopsis genome contains three *RACK1 *homologous genes, designated as *RACK1A*, *RACK1B *and *RACK1C*, respectively, which encode three highly similar proteins. *RACK1 *genes regulate plant development likely in a continuous quantitative manner. *RACK1A *is likely the ancestral gene whereas *RACK1B *and *RACK1C *are the duplicate genes, because *RACK1A *retains the most functions of *RACK1 *genes. The expression of *RACK1 *follows a general trend of *RACK1A *> *RACK1B *> *RACK1C*. A certain threshold of gene activity is likely required for the *RACK1 *genes to have any influence on plant development, and the gene activity can be saturated once an excess of gene activity is reached. Because the loss-of-function mutations in *RACK1B *or *RACK1C *or both do not confer any defects in plant development while enhancing the developmental defects of *rack1a *mutants, the residual activities of *RACK1B *and *RACK1C *are likely above this threshold but below the point of saturation. *RACK1 *genes mutually regulate each other's transcription. Both the difference in gene expression and the cross-regulation are likely the molecular determinants of unequal genetic redundancy of *RACK1 *genes in regulating plant development. The model is schematically based on the possible explanations for unequal genetic redundancy provided by Briggs et al. (2006) [16].

Among three *RACK1 *homologous genes in Arabidopsis, *RACK1A *is likely the ancestral gene whereas *RACK1B *and *RACK1C *are duplicate genes because *RACK1A *appears to retain most of the function of *RACK1 *gene family. *RACK1 *genes regulate plant development in a continuous, quantitative manner. It is likely that a certain threshold of gene activity is required for the *RACK1 *genes to have any influence on the plant development (Figure 8). Because *rack1b *and *rack1c *single mutants do not exhibit any defects in plant development whereas the *rack1a rack1b *and *rack1a rack1c *double mutants display enhanced phenotypes compared with the *rack1a *single mutant, it is likely that the residual activities of *RACK1B *and *RACK1C *are above this threshold (Figure 8). Therefore, although both *RACK1B *and *RACK1C *are likely dispensable, they still contribute significantly to the overall activity of *RACK1 *genes. Both the difference in gene expression level and the cross-regulation are likely the molecular determinants of unequal genetic redundancy of *RACK1 *genes in regulating plant development.

## Methods

### Plant materials and growth conditions

All mutants are in the Arabidopsis Columbia (Col-0) ecotype background. The *rack1a-1 *and *rack1a-2 *mutants have been reported previously [15]. Plants were grown in 5 × 5 cm pots containing moistened 1 : 3 mixture of Sunshine Mix #1 (Sun Gro Horticulture Canada Ltd., ) and Metro-Mix 220 (W.R. Grace & Co., ) with 10/14 h (short-day conditions) or 14/10 h (long-day conditions) photoperiod at approximately 120 μmol m^-2 ^s^-1 ^at 23°C.

### Isolation of *rack1b *and *rack1c *T-DNA insertional mutants

The T-DNA insertion mutants of *RACK1B *(At1g48630), *rack1b-1 *(SALK_117422) and *rack1b-2 *(SALK_145920), and the T-DNA insertion mutants of *RACK1C *(At3g18130), *rack1c-1 *(SAIL_199_A04) and *rack1c-2 *(SALK_017913), were identified from the SALK T-DNA Express database . For the SALK T-DNA insertional mutants [20], the insertion was confirmed by PCR using *RACK1B*-specific primers (5'-TCTCGACCTCAAACCCTG-3' and 5'-GAGAAGACTTTAGAGTCGATGGA-3') or *RACK1C*-specific primers (5'-ATCTCTCGCTCTGTTACGC-3' and 5'-ACAATACTGACGCAGTCTGG-3') and a T-DNA left border-specific primer JMLB1 (5'-GGCAATCAGCTGTTGCCCGTCTCACTGGTG-3'). For the SAIL T-DNA insertion mutants [21], a different T-DNA left border-specific primer, GarlicLB3 (5'-TAGCATCTGAATTTCATAACCAATCTCGATACAC-3'), was used. The absence of full-length transcript of *RACK1B *or *RACK1C *in these alleles was confirmed by RT-PCR.

### Generation of *rack1a*, *rack1b *and *rack1c *double and triple mutants

Double mutants between *rack1a-1 *and *rack1b-2 *or *rack1c-1 *were generated by crossing *rack1b-2 *or *rack1c-1 *into *rack1a-1 *single mutant and isolated in the F2 progeny by PCR genotyping. Similarly, double mutants between *rack1b-2 *and *rack1c-1 *were generated by crossing *rack1c-1 *into *rack1b-2 *single mutant and isolated in the F2 progeny by PCR genotyping. For simplicity, the *rack1a rack1b*, *rack1a rack1c *and *rack1b rack1c *double mutant nomenclatures in this report refer specifically to the *rack1a-1 rack1b-2*, *rack1a-1 rack1c-1 *and *rack1b-2 rack1c-1 *mutants, respectively.

Triple mutant among *rack1a-1*, *rack1b-2 *and *rack1c-1 *was generated by crossing *rack1b-2 rack1c-1 *into *rack1a-1 rack1b-2 *double mutants. Because *rack1a-1 rack1b-2 rack1c-1 *triple mutants cannot survive in soil to maturity, they are maintained in plants homozygous for the *rack1b-2 *and *rack1c-1 *loci and heterozygous for the *rack1a-1 *locus. The status of triple mutant was confirmed by PCR genotyping.

### Genetic complementation

The full-length open-reading frames of *RACK1A *(At1g18080), *RACK1B *and *RACK1C *were amplified from a cDNA library made from seedlings grown in light for 10 d, cloned into the pENTR/D-TOPO vector (Invitrogen, ), and then subcloned into Gateway plant transformation destination binary vector pB2GW7 [22] by LR recombination reactions. In these constructs, the expression of *RACK1A*, *RACK1B *or *RACK1C *was driven by the *35S *promoter of the *Cauliflower mosaic virus*. Binary vectors were transformed into *rack1a-1 *or *rack1a-2 *mutants by *Agrobacterium*-mediated transformation [23]. At least 16 independent transgenic lines were selected from each transformation, and two to four representative lines were used for further studies. The expression of transgene was examined by RT-PCR.

### RNA isolation, RT-PCR and quantitative real-time PCR analyses

For tissue/organ expression pattern analysis, total RNA was isolated from different parts of seedlings or mature plants, using the TRIzol reagent (Invitrogen). cDNA was synthesized from 1 μg total RNA by oligo(dT)_20_-primed reverse transcription, using THERMOSCRIPT RT (Invitrogen). *RACK1A*-specific primers (5'-GGCATCTCCAGACACCGAAA-3' and 5'-GCAGAGAGCAACGACAGC-3'), *RACK1B*-specific primers (5'-TCTCGACCTCAAACCCTG-3' and 5'-GAGAAGACTTTAGAGTCGATGGA-3'), and *RACK1C*-specific primers (5'-ATCTCTCGCTCTGTTACGC-3' and 5'-ACAATACTGACGCAGTCTGG-3') were used to amplify the transcripts of these three genes, respectively. The expression of *ACTIN2 *(amplified by primers 5'-GTTGGGATGAACCAGAAGGA-3' and 5'-GAACCACCGATCCAGACACT-3') was used as a control in PCR reactions. For the examination of the transcript level of *RACK1A*, *RACK1B *and *RACK1C *in the T-DNA insertional mutants or in the transgenic lines, 10 d-old, light-grown seedlings were used for total RNA isolation.

For the quantitative analysis of *RACK1A*, *RACK1B *and *RACK1C *transcript levels in the different tissues/organs of wild-type Col plants or in the *rack1a-1*, *rack1b-2 *and *rack1c-1 *single and double mutants, real-time PCR was performed. *RACK1A*-specific real-time PCR primers (5'-CTGAGGCTGAAAAGGCTGACAACAG-3' and 5'-CTAGTAACGACCAATACCCCAAACTC-3'), *RACK1B*-specific real-time PCR primers (5'-GGTTCTACTGGAATCGGAAACAAGACC-3' and 5'-CTAGTAACGACCAATACCCCAGACCC-3'), and *RACK1C*-specific real-time PCR primers (5'-GCAGAGAAGAATGAAGGTGGTGT-3' and 5'-CTAGTAACGACCAATACCCCAGACCC-3') were used. The expression of *ACTIN2 *(amplified by real-time PCR primers 5'-CCAGAAGGATGCATATGTTGGTGA-3'and 5'-GAGGAGCCTCGGTAAGAAGA-3') was used to normalize the expression of each gene. The quantitative real-time PCR was performed using the MJ MiniOpticon real-time PCR system (Bio-Rad, ) and IQ SYBR Green Supermix (Bio-Rad).

### Rosette leaf production assay

The number of rosette leaves was collected from wild-type Col and mutant plants grown under 10/14 h or 14/10 h photoperiod with approximately 120 μmol m^-2 ^s^-1 ^at 23°C. At least four plants from each genotype were used in each experiment, and the experiment was repeated twice. The rate of rosette leaf production was expressed as the number of rosette leaves divided by the age of plant.

### Root growth assay

Seedlings were grown on MS/G plates consisting of 1/2 Murashige & Skoog (MS) basal medium supplemented with vitamins (Plantmedia, ), 1% (w/v) sucrose and 0.6% (w/v) phytoagar (Plantmedia), with pH adjusted to 5.7 with 1N KOH. The plates were placed under 14/10 h photoperiod with approximately 120 μmol m^-2 ^s^-1 ^at 23°C with a vertical orientation for monitoring root growth. The length of primary and the number of lateral roots were collected from at least 15 seedlings each genotype.

## Authors' contributions

JG isolated the *rack1 *single, double and triple mutants, and conducted all experiments. JGC conceived of the study and participated in its design and coordination. All authors participated in drafting and editing the manuscript, and read and approved the final manuscript.

## References

[B1] Mochly-Rosen D, Khaner H, Lopez J (1991). Identification of intracellular receptor proteins for activated protein kinase C. Proc Natl Acad Sci USA.

[B2] Ron D, Chen CH, Caldwell J, Jamieson L, Orr E, Mochly-Rosen D (1994). Cloning of an intracellular receptor for protein kinase C – A homolog of the β-subunit of G-proteins. Proc Natl Acad of Sci USA.

[B3] McCahill A, Warwicker J, Bolger GB, Houslay MD, Yarwood SJ (2002). The RACK1 scaffold protein: A dynamic cog in cell response mechanisms. Mol Pharmacol.

[B4] Sklan EH, Podoly E, Soreq H (2006). RACK1 has the nerve to act: structure meets function in the nervous system. Prog Neurobiol.

[B5] Ishida S, Takahashi Y, Nagata T (1993). Isolation of cDNA of an auxin-regulated gene encoding a G-protein β-subunit-like protein from tobacco BY-2-cells. Proc Natl Acad Sci USA.

[B6] Guo J, Liang J, Chen JG (2007). RACK1: a versatile scaffold protein in plants?. Int J Plant Dev Biol.

[B7] McKhann HI, Frugier F, Petrovics G, delaPena TC, Jurkevitch E, Brown S, Kondorosi E, Kondorosi A, Crespi M (1997). Cloning of a WD-repeat-containing gene from alfalfa (*Medicago sativa*): a role in hormone-mediated cell division?. Plant Mol Biol.

[B8] Perennes C, Glab N, Guglieni B, Doutriaux MP, Phan TH, Planchais S, Bergounioux C (1999). Is arcA3 a possible mediator in the signal transduction pathway during agonist cell cycle arrest by salicylic acid and UV irradiation?. J Cell Sci.

[B9] Iwasaki Y, Komano M, Ishikawa A, Sasaki T, Asahi T (1995). Molecular cloning and characterization of cDNA for a rice protein that contains seven repetitive segments of the Trp-Asp forty-amino-acid repeat (WD-40 repeat). Plant Cell Physiol.

[B10] Komatsu S, Abbasi F, Kobori E, Fujisawa Y, Kato H, Iwasaki Y (2005). Proteomic analysis of rice embryo: an approach for investigating Gα protein-regulated proteins. Proteomics.

[B11] Nakashima A, Chen L, Thao NP, Fujiwara M, Wong HL, Kuwano M, Umemura K, Shirasu K, Kawasaki T, Shimamoto K (2008). RACK1 functions in rice innate immunity by interacting with the Rac1 immune complex. Plant Cell.

[B12] Chang IF, Szick-Miranda K, Pan S, Bailey-Serres J (2005). Proteomic characterization of evolutionarily conserved and variable proteins of Arabidopsis cytosolic ribosomes. Plant Physiol.

[B13] Giavalisco P, Wilson D, Kreitler T, Lehrach H, Klose J, Gobom J, Fucini P (2005). High heterogeneity within the ribosomal proteins of the *Arabidopsis thaliana *80S ribosome. Plant Mol Biol.

[B14] Ullah H, Scappini EL, Moon AF, Williams LV, Armstrong DL, Pedersen LC (2008). Structure of a signal transduction regulator, RACK1, from *Arabidopsis thaliana*. Protein Sci.

[B15] Chen JG, Ullah H, Temple B, Liang J, Guo J, Alonso JM, Ecker JR, Jones AM (2006). RACK1 mediates multiple hormone responsiveness and developmental processes in Arabidopsis. J Exp Bot.

[B16] Briggs GC, Osmont KS, Shindo C, Sibout R, Hardtke CS (2006). Unequal genetic redundancies in Arabidopsis – a neglected phenomenon?. Trends Plant Sci.

[B17] Kempin SA, Savidge B, Yanofsky MF (1995). Molecular basis of the *cauliflower *phenotype in *Arabidopsis*. Science.

[B18] Holm M, Ma LG, Qu LJ, Deng XW (2002). Two interacting bZIP proteins are direct targets of COP1-mediated control of light-dependent gene expression in Arabidopsis. Genes Dev.

[B19] Caño-Delgado A, Yin Y, Yu C, Vafeados D, Mora-García S, Cheng JC, Nam KH, Li J, Chory J (2004). BRL1 and BRL3 are novel brassinosteroid receptors that function in vascular differentiation in *Arabidopsis*. Development.

[B20] Alonso JM, Stepanova AN, Leisse TJ, Kim CJ, Chen H, Shinn P, Stevenson DK, Zimmerman J, Barajas P, Cheuk R, Gadrinab C, Heller C, Jeske A, Koesema E, Meyers CC, Parker H, Prednis L, Ansari Y, Choy N, Deen H, Geralt M, Hazari N, Hom E, Karnes M, Mulholland C, Ndubaku R, Schmidt I, Guzman P, Aguilar-Henonin L, Schmid M, Weigel D, Carter DE, Marchand T, Risseeuw E, Brogden D, Zeko A, Crosby WL, Berry CC, Ecker JR (2003). Genome-wide insertional mutagenesis of *Arabidopsis thaliana*. Science.

[B21] Sessions A, Burke E, Presting G, Aux G, McElver J, Patton D, Dietrich B, Ho P, Bacwaden J, Ko C, Clarke JD, Cotton D, Bullis D, Snell J, Miguel T, Hutchison D, Kimmerly B, Mitzel T, Katagiri F, Glazebrook J, Law M, Goff SA (2002). A high-throughput Arabidopsis reverse genetics system. Plant Cell.

[B22] Karimi M, Inze D, Depicker A (2002). GATEWAY((TM)) vectors for *Agrobacterium*-mediated plant transformation. Trends Plant Sci.

[B23] Clough SJ, Bent AF (1998). Floral dip: a simplified method for *Agrobacterium*-mediated transformation of *Arabidopsis thaliana*. Plant J.

